# ‘The Reports of My Death Are Greatly Exaggerated’—Evaluating the Effect of Necrosis on *MGMT* Promoter Methylation Testing in High-Grade Glioma

**DOI:** 10.3390/cancers16101906

**Published:** 2024-05-16

**Authors:** Laveniya Satgunaseelan, Maggie Lee, Sebastian Iannuzzi, Susannah Hallal, Kristine Deang, Kristian Stanceski, Heng Wei, Sofia Mason, Brindha Shivalingam, Hao-Wen Sim, Michael E. Buckland, Kimberley L. Alexander

**Affiliations:** 1 Department of Neuropathology, Royal Prince Alfred Hospital, Camperdown, NSW 2050, Australia; maggie.lee@sydney.edu.au (M.L.); sebastiannicholas.iannuzzi@health.nsw.gov.au (S.I.); susannah.hallal@lh.org.au (S.H.); kristian.stanceski@sydney.edu.au (K.S.); heng.wei@sydney.edu.au (H.W.); michael.buckland@sydney.edu.au (M.E.B.); kim.alexander@lh.org.au (K.L.A.); 2 Faculty of Medicine and Health, School of Medicine, University of Sydney, Camperdown Campus, Sydney, NSW 2000, Australia; kristine.deang@lh.org.au (K.D.); brindha@brain-surgeon.com.au (B.S.); 3 Department of Neurosurgery, Chris O’Brien Lifehouse, Camperdown, NSW 2050, Australia; 4 Department of Medical Oncology, Chris O’Brien Lifehouse, Camperdown, NSW 2050, Australia; sofia.mason@lh.org.au (S.M.); haowen.sim@lh.org.au (H.-W.S.); 5 Garvan Institute of Medical Research, Darlinghurst, NSW 2010, Australia; 6 Faculty of Medicine and Health, University of New South Wales, Sydney, NSW 2052, Australia; 7 Department of Neurosurgery, Royal Prince Alfred Hospital, Camperdown, NSW 2050, Australia; 8 NHMRC Clinical Trials Centre, University of Sydney, Camperdown, NSW 2050, Australia; 9 Department of Medical Oncology, The Kinghorn Cancer Centre, Darlinghurst, NSW 2010, Australia

**Keywords:** glioma, glioblastoma, astrocytoma, *MGMT* promoter, necrosis

## Abstract

**Simple Summary:**

The *MGMT* gene is responsible for repairing DNA damage, including as a result of chemotherapy, and, therefore, antagonizes its effects. If the *MGMT* gene is ‘silenced’, there is defective DNA repair leading to increased chemotherapy-related tumor cell death. *MGMT* gene silencing can occur through a process called ‘promoter methylation’. Specifically, the measurement of *MGMT* promoter methylation (*MGMT*p testing) has proven to be a robust way to predict which brain tumor patients will respond to chemotherapy. However, prior to treatment, brain tumors can already contain variably sized areas of dead tumor termed ‘necrosis’. Necrosis has traditionally been assumed to reduce the reliability of *MGMT*p testing but has not been previously investigated. In this study, we demonstrate that necrosis has no material effect on the results of *MGMT*p testing, thus allowing for the inclusion of a broader range of brain tumor samples for future analysis.

**Abstract:**

(1) Background: *MGMT* (O-6-methylguanine-DNA methyltransferase) promoter methylation remains an important predictive biomarker in high-grade gliomas (HGGs). The influence of necrosis on the fidelity of *MGMT* promoter (*MGMT*p) hypermethylation testing is currently unknown. Therefore, our study aims to evaluate the effect of varying degrees of necrosis on *MGMT*p status, as determined by pyrosequencing, in a series of primary and recurrent HGGs; (2) Methods: Within each case, the most viable blocks (assigned as ‘true’ MGMTp status) and the most necrotic block were determined by histopathology review. *MGMT*p status was determined by pyrosequencing. Comparisons of *MGMT*p status were made between the most viable and most necrotic blocks. (3) Results: 163 samples from 64 patients with HGGs were analyzed. *MGMT*p status was maintained in 84.6% of primary and 78.3% of recurrent HGGs between the most viable and necrotic blocks. A threshold of ≥60% tumor cellularity was established at which *MGMT*p status was unaltered, irrespective of the degree of necrosis. (4) Conclusions: *MGMT*p methylation status, as determined by pyrosequencing, does not appear to be influenced by necrosis in the majority of cases at a cellularity of at least 60%. Further investigation into the role of intratumoral heterogeneity on *MGMT*p status will increase our understanding of this predictive marker.

## 1. Introduction

Despite vigorous efforts to broaden treatment options for high-grade gliomas (HGGs) [1,2], surgery, radiotherapy and temozolomide (TMZ)-based regimens remain the mainstay of therapy for adult HGGs [3]. Given its universally dismal prognosis, whether IDH-wildtype, IDH-mutant or histone H3 mutant, the prediction of tumor response to TMZ plays a key role in clinical decision-making [4,5,6,7]. For over a decade, the assessment of *MGMT* (O-6-methylguanine-DNA methyltransferase) promoter methylation has been an important predictive and prognostic biomarker in neuro-oncology, guiding the current standard of care. In the IDH-wildtype glioblastoma (GBM IDHwt) setting, hypermethylation of the *MGMT* promoter region has shown a consistent association with improved survival in response to TMZ [8,9,10,11,12]. This is of particular importance in selecting elderly patients for TMZ therapy, in whom TMZ-related toxicities are less well tolerated, and the benefit in *MGMT*p unmethylated patients is much less pronounced, if present at all [9,11,13]. Given the hypermethylated phenotype of IDH-mutant astrocytomas, *MGMT* promoter hypermethylation is prevalent in IDH-mutant astrocytomas; however, it is said to have prognostic, as opposed to predictive, value [14,15]. Similar findings have been made in diffuse hemispheric glioma, H3 G34-mutant [6,16]. In the work-up of any adult-onset glioma, *MGMT* promoter methylation testing is a frequent theranostic adjunct.

*MGMT*, located at 10q26.3 and containing five exons, encodes the MGMT enzyme, which is vital for the repair of DNA damaged by alkylating agents, including TMZ [17]. Alkylating agents cause the addition of alkyl adducts at the O^6^ position of guanine, which unchecked by MGMT, results in base mispairing (with thymine), futile DNA repair cycles by mismatch repair proteins, eventual cell cycle arrest and cell death [17,18]. MGMT identifies and removes alkyl adducts at the O^6^ position of guanine, thus reversing the effects of alkylating agents while itself becoming inactivated [17,19]. Transcriptional silencing of *MGMT*, which occurs largely by promoter hypermethylation [20,21], therefore, enhances the response to TMZ by reducing the efficacy of DNA alkylation repair [22]. 

The promoter region and exon 1 of *MGMT* contain a CpG island, which is 777 bp long and contains 98 CpG dinucleotides [23,24]. The methylation status of two different regions within the CpG island has an established significant correlation with *MGMT* mRNA expression, referred to as differentially methylated region 1 (DMR1; CpG25-50) and differentially methylated region 2 (DMR2; CpG73-90) [25]. DMR2 arose as the critical region for *MGMT* promoter methylation testing, as mutagenic substitutions of CpG sites within DMR2 resulted in reduced *MGMT* promoter activity, and DMR2 was consistently found to be methylated when DMR1 was methylated [21,25]. DMR2 is located within exon 1 of the *MGMT* gene, and testing of this region reliably correlates with *MGMT* gene silencing, as demonstrated using a variety of methods over the years [22,25,26].

A consensus method for *MGMT* promoter region testing has not yet been established [23], and the detection assays used include methylation-specific polymerase chain reaction (MSP), pyrosequencing and Infinium Methylation EPIC BeadChip Arrays [23,26,27]. Most techniques rely on the bisulfite treatment of DNA (resulting in the conversion of unmethylated cytosines to uracils) and, therefore, the identification of methylated and unmethylated CpG sites [28]. While MSP was used in the initial clinical trials, it has subsequently been shown to have equivocal replicability and poor performance in formalin-fixed paraffin-embedded (FFPE) tissue [23,29]. EPIC arrays provide more coverage of CpG sites, including good coverage of *MGMT*; however, they remain costly and time-intensive assays to implement in the clinical diagnostic setting [26,30]. Pyrosequencing gives a quantitative picture of individual CpG sites within *MGMT* [27,28,31], as well as being a relatively cheap assay that is amenable to application in a high throughput setting. *MGMT* promoter methylation analysis by pyrosequencing has been repeatedly shown to be the most sensitive method for clinical use [27,28,31] and a robust prognostication tool [27,28,31,32,33,34,35,36]. 

The lack of a consensus method has meant that established cut-offs for defining a methylated versus unmethylated *MGMT* promoter region are yet to be definitively evaluated in a large clinical trial [23]. Nevertheless, on review of the literature, the cut-off for unmethylated cases is cited between 7 and 9% [27,32,33,36,37], with variable thresholds given for methylated cases [32,33,36,37]. 

Given the absence of agreement on the optimal methodology and cut-offs within the analytical phase of *MGMT* promoter methylation testing [15,23], it is, therefore, vital to attempt to standardize pre-analytical factors [38]. Tumor viability is an important pre-analytical factor; however, it can be difficult to optimize in high-grade gliomas due to the variable presence of necrosis [39]. Currently, there are no guidelines on the thresholds for necrosis in the selection of FFPE tissue for solid tumor testing. Frequently, significantly necrotic tumor material may be all that is available for clinical testing; however, there are little data in the literature as to whether this tissue should be tested, and if so, whether the results are trustworthy. Given that necrosis is one of the defining histological features of HGGs, our study sought to evaluate the effect of varying degrees of necrosis on the fidelity of *MGMT* promoter (*MGMT*p) methylation test results. From this, we aimed to establish thresholds for tumor necrosis for *MGMT*p testing to aid pathologists in selecting appropriate tissue for molecular work-up. We also examined whether *MGMT*p methylation status switches between matched primary and recurrent HGGs and their relationship to clinical outcome parameters. 

## 2. Materials and Methods

### 2.1. Case Selection

The study was conducted under institutional ethics approval (2019/ETH08929). Selection criteria included (1) patient age over 18 years; (2) primary HGG diagnosis made by central pathology review at the Department of Neuropathology, Royal Prince Alfred Hospital (RPAH); and (3) the availability of slides and blocks for histopathology review. Where the selection criteria were met, methylated *MGMT*p cases were included preferentially so that the effect of necrosis on the methylation percentage by pyrosequencing could be assessed.

We identified 85 primary and recurrent cases from 64 patients consented to the Sydney Brain Tumour Bank over a period from 2012 to 2024, with slides and blocks available for review. Four patients who had matched primary and recurrent tumors (overall, seven cases) did not have blocks available for repeat testing of the most necrotic block but were included for analysis of potential *MGMT*p methylation status switch at recurrence; therefore, 163 blocks were tested in total. 

### 2.2. Histopathology Review

All cases were reviewed by specialist neuropathologists (LS and MEB) and appropriately designated according to the current WHO Classification of Tumors of the Central Nervous System [40]. Hematoxylin and eosin (H&E) stained sections from each case were reviewed to identify the FFPE blocks with the least and the most necrosis in each case. The degree of necrosis was determined by assessing the necrotic tumor as a proportion of the overall tumor cellularity (both necrotic and viable). The least necrotic or most viable tumor block was referred to as ‘VT’, and the most necrotic tumor block was referred to as ‘NT’. 

### 2.3. MGMT Promoter Methylation Testing

*MGMT*p testing was performed using a standard, clinically validated laboratory pyrosequencing assay. DNA was extracted from scrolls obtained from the FFPE blocks. DNA extractions were performed using a QIAamp DNA FFPE Tissue Kit (Qiagen, Hilden, Germany). Bisulfite treatment of the DNA was undertaken using an EpiTect Bisulfite Kit (Qiagen). Pyrosequencing of the *MGMT*p region was undertaken using a PyroMark therascreen MGMT kit (Qiagen) and the PyroMark Q24 system (Qiagen), which detect four CpG sites located in exon 1. The mean of the methylation percentages at each CpG site was then used to determine methylation status. Cut-offs determined by our laboratory for *MGMT*p methylation status are as follows: <9%—unmethylated, ≥9% and ≤13%—borderline, and >13% methylated. The borderline zone was calculated using clinically validated measurement uncertainty calculations [41]. Cases designated as ‘borderline’ are generally treated as methylated in the clinical setting.

### 2.4. Statistical Analysis

Statistical significance was set at *p*  < 0.05. Comparisons between median *MGMT*p methylation percentages were performed using Mann–Whitney U tests. We used repeated measures correlation (rmcorr) analyses using the rmcorrShiny app (https://lmarusich.shinyapps.io/shiny_rmcorr/, accessed on 16 March 2024) to calculate within-individual associations of different levels of tumor necrosis (%) and repeated assessments of *MGMT*p methylation in a total of 163 tumor specimens (primary and recurrences) [42,43]. Spearman’s rank correlation coefficient was used to compare methylation percentages between different levels of tumor cellularity and necrosis. Although the treatment details and survival outcomes were available (Supplementary Table S1), survival analyses were not performed due to the deliberate selection bias toward methylated *MGMT*p cases in order to assess the effect of necrosis. 

## 3. Results

### 3.1. Study Cohort Characteristics

Sixty-four patients were included in the study, with a median age of 60 years and a male preponderance. The vast majority of cases were of glioblastoma, IDH-wildtype, with other high-grade subtypes also included. Diffuse astrocytic gliomas, which were histologically grade 3 and harbored *TERT* promoter variants, were included as comparison controls to assess for intratumoral heterogeneity. Clinical information was available for 61 patients. The patient characteristics are summarized in Table 1; complete demographic, clinicopathologic and outcome information is available in Supplementary Table S1.

**Figure 1:**
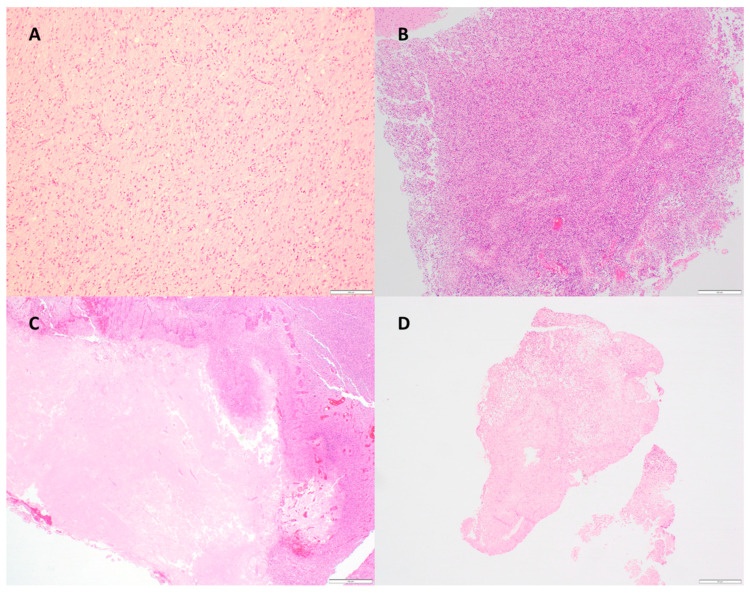
H&E sections demonstrating (**A**) diffuse astrocytic gliomas, histologically grade 3 with molecular feature of GBM (*TERT* promoter variant) (100× magnification); (**B**) glioblastoma, IDH-wildtype, with 90% tumor cellularity/10% necrosis (40× magnification); (**C**) glioblastoma, IDH-wildtype, with 90% tumor cellularity/60% necrosis (40× magnification); (**D**) glioblastoma, IDH-wildtype, with 90% tumor cellularity/90% necrosis (20× magnification).

Overall, 163 blocks from 64 patients were analyzed. Of the VT blocks, the median tumor cellularity was 80% (range 50–95%), and the median tumor necrosis was 0% (range 0–70%). Of the NT blocks, the median tumor cellularity was 90% (range 60 to 95%), and the median tumor necrosis was 30% (range 5 to 90%). Figure 1 demonstrates examples of different degrees of tumor necrosis as assessed by H&E sections.

### 3.2. The Effect of Necrosis on MGMTp Status in Primary Presentations of High-Grade Glioma

Fifty-eight patients with primary HGG resections were included in the study. The ‘molecular GBM’ (*n* = 4) group, by definition, lacked necrosis and was analyzed separately. Two primary resection cases did not have an NT block available for testing, allowing for the analysis of 52 cases.

**Figure 2:**
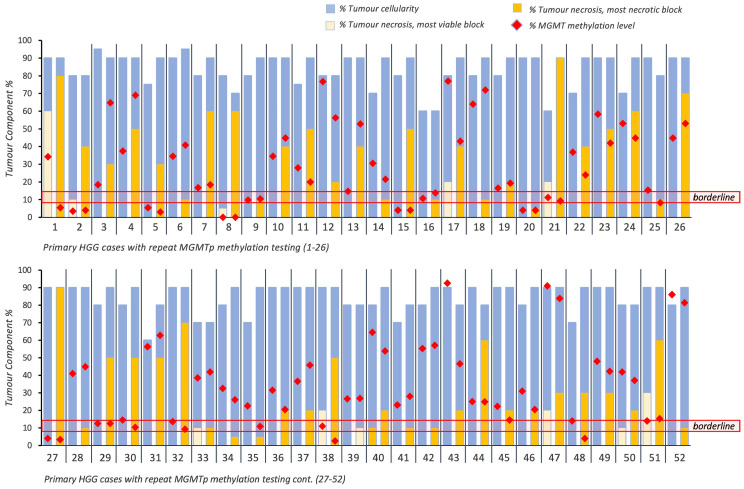
Overview of intratumoral cellularity, necrosis and *MGMT*p methylation percentages across 52 primary HGG cases with repeated *MGMT*p testing. Horizontal red lines indicate the borderline *MGMT*p zone (9–13%).

Of 52 patients (Figure 2), 44 patients (84.6%) maintained their *MGMT*p status regardless of necrosis. No evident differences in *MGMT*p percentages were observed between the VT and NT blocks, irrespective of *MGMT*p status (*p* > 0.05; Table 2).

*MGMTp* status changed in eight patients (15.4%) regardless of the presence of necrosis. Changes around the borderline cut-off zone of 13% were noted in five cases. These included borderline *MGMT*p status in the VT block (10.8%) vs. methylated in the NT block (13.8%; *n* = 1) and methylated *MGMT*p in the VT block (14.5–22.5%) vs. borderline *MGMT*p in the NT block (8.3–10.8%; *n* = 4). The remaining three cases changed from borderline (*n* = 2) or methylated *MGMT*p (*n* = 1) status (VT block, 0 to 60% necrosis) to unmethylated (NT blocks comprising 30 to 80% necrosis; Figure 2 and Supplementary Table S1). 

Given that changes in MGMTp status in five patients between borderline and methylated *MGMT*p status would have not affected the clinical translation of the result (i.e., ‘methylated’), we conclude that in 94.2% of primary HGGs (49 cases), the degree of necrosis did not materially influence *MGMT*p status. 

### 3.3. The Effect of Necrosis on MGMTp Status in Recurrent Presentations of High-Grade Glioma

**Figure 3:**
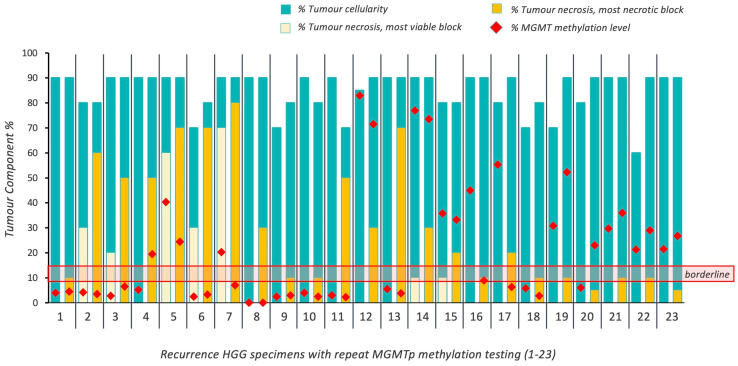
Overview of intratumoral cellularity, necrosis and *MGMT*p methylation percentages in 23 cases of recurrent HGG. Horizontal red lines indicate the borderline *MGMT*p zone (9–13%).

Twenty-three patients with 27 resections of recurrent HGG were included in the study. Four recurrent resection cases did not have an NT block available for testing. Of the available 23 cases (Figure 3), 18 patients (78.3%) maintained their *MGMT*p status regardless of necrosis. Again, no evident differences in *MGMT*p percentages were observed, regardless of *MGMT*p status (*p* > 0.05; Table 2). 

In the recurrence setting, five patients (21.7%) did not maintain their *MGMT*p status on repeat testing, irrespective of the degree of tumor necrosis. One case changed from methylated (45% *MGMT*p methylation level) to borderline (9%), with 0 and 10% necrosis in the respective VT and NT blocks. Two cases changed from methylated (20.3% and 55%) to unmethylated (7% and 6.3%), where the levels of necrosis in the NT blocks were 20% and 90%, respectively. Two cases changed from unmethylated (5.25% and 6%) to methylated (40.3% and 23%), with 5% and 60% necrosis in each of the NT blocks (Figure 3; Supplementary Table S1). The majority of cases of recurrent HGG demonstrated no effect of necrosis; however, bidirectional changes in *MGMT*p status were observed in some recurrent cases at varying degrees of necrosis.

### 3.4. Establishing Thresholds for Tumor Cellularity and Degrees of Necrosis in MGMTp Methylation Testing

**Figure 4:**
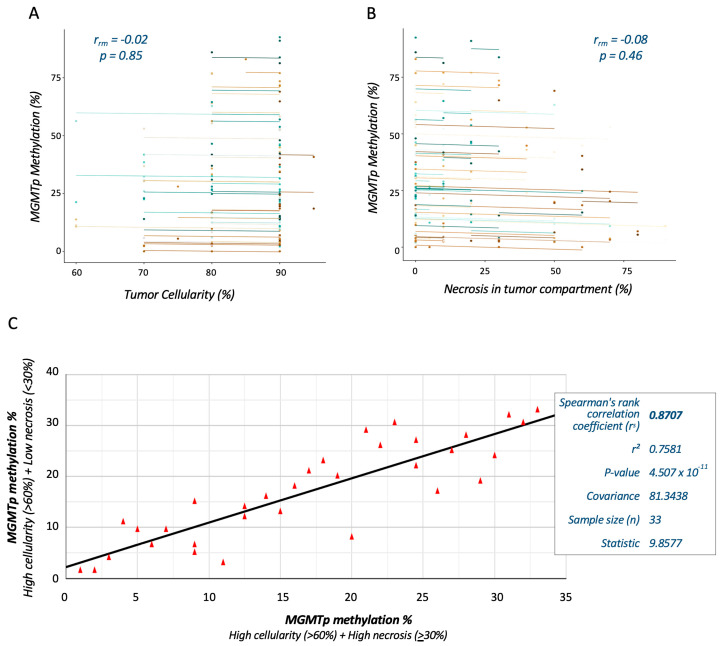
Correlation analyses demonstrate the reproducibility of *MGMT*p methylation testing in HGG sections with varying degrees of tumor necrosis. Repeated measures correlation analysis shows no significant association between *MGMT*p status and tumor cellularity (**A**) or necrosis (**B**). Each participant, plotted in a different color, contributes two paired data points representing repeated *MGMT*p tests on different tissue blocks. The corresponding lines depict the repeated measures correlation model. The relationship of (black) line-of-best-fit between *MGMT*p methylation percentages (data points depicted by red triangles) at thresholds of 30% necrosis and 60% tumor cellularity was determined by Spearman rank correlation (**C**).

The overall relationships between tumor cellularity and the extent of necrosis with *MGMT*p methylation testing were assessed in VT and NT blocks. Using repeated measures correlation analyses, no significant associations between *MGMT*p methylation percentages and tumor cellularity (r_rm_ = 0.02; *p* = 0.85) or necrosis (r_rm_ = −0.08; *p* = 0.46) were observed (Figure 4A,B). 

We further explored the reproducibility of *MGMT*p methylation testing by comparing VT and NT blocks from the same cases. Cases with ≥60% tumor cellularity in all blocks, <30% necrosis in the VT block and ≥30% necrosis in the NT block were selected for analysis (total *n* = 33). Spearman rank correlation analysis demonstrated a strong monotonic relationship (r_s_ = 0.76; *p* < 0.001) between the *MGMT*p methylation percentages measured from VT and NT blocks at thresholds of 30% necrosis and 60% tumor cellularity (Figure 4C).

A more granular examination of *MGMT*p status changes by degree of necrosis is summarized in Table 3. Although our diagnostic laboratory reports borderline and methylated MGMTp status, both are generally treated as ‘methylated’ in the clinical setting. Therefore, detecting an *MGMT*p status change from borderline/methylated to unmethylated and vice versa would represent a clinically significant finding. In primary HGGs, the majority of *MGMT*p status changes were from a higher to lower methylated status (*n* = 5); however, this was usually insufficient to change the reportable *MGMT*p methylation status. Three cases are noted to change from borderline/methylated to unmethylated *MGMT*p amongst the primary cases, where NT blocks contain at least 30% necrosis. In recurrent HGG, changes are noted in both directions (high to low methylated *MGMT*p status and vice versa) across the varying degrees of necrosis. No association with age, gender or treatment was observed. All patients with an *MGMT*p status change completed chemoradiotherapy. Survival data were only available for six patients, making it difficult to make meaningful comparisons between the groups.

### 3.5. Changes in MGMTp Status in Less Common HGG Subtypes

Two cases of IDH-mutant astrocytoma and one case of diffuse hemispheric glioma, H3 G34-mutant were included in the cohort. The IDH-mutant astrocytomas were each borderline (*MGMT*p methylation percentage, 14%) and methylated (45%), with both changing on testing to unmethylated (4%) and borderline (9%), respectively. The case of DHG, H3 G34 maintained a methylated *MGMT*p status across the VT (18.5%) and NT (64.8%) blocks.

Three cases of ‘molecular GBM’, all without necrosis by definition, were tested across two different blocks; however, they were not included in the analysis of the effect of necrosis on *MGMT*p status. (The fourth case did not have blocks available for repeat testing but was included for the assessment of *MGMT*p status switches between primary and recurrence.) One case changed from a methylated (14.8%) to unmethylated (2%) *MGMT*p status despite having the same tumor cellularity (90%) in two different blocks. The same result was confirmed on repeat testing. The other two cases maintained their *MGMT*p status. 

### 3.6. Evaluation of MGMTp Status Switches between Matched Primary and Recurrent HGGs

**Figure 5:**
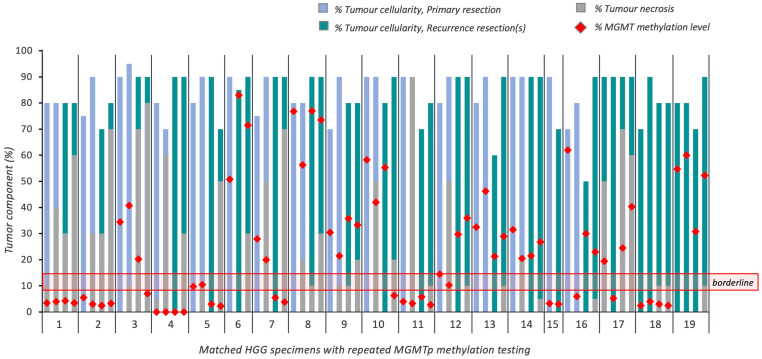
Overview of tumoral cellularity, necrosis and *MGMT*p methylation percentages in 19 cases of recurrent HGG (16 recurrences; 3 re-resected recurrences). Horizontal red lines indicate the borderline *MGMT*p zone (9–13%).

Primary and recurrence tumor pairs were tested from 16 patients, in which one primary ‘molecular GBM’ was included (primary VT blocks—70–90% cellularity, 0–10% necrosis; primary NT blocks—70–95% cellularity; 0–90% necrosis) (Figure 5). Additionally, we tested three patients with multiple re-resected recurrences who underwent primary resections at other institutions (VT blocks at initial testing at our institution—70–90% cellularity, 0–50% necrosis; NT blocks—80–90% cellularity; 10–70% necrosis) (Figure 5).

Of the 16 matched primary and recurrent tumors, ten cases had methylated *MGMT*p, one was in the borderline zone, and five were unmethylated. Maintenance of *MGMT*p methylation status was seen in 13 cases (81.3%).

*MGMT*p methylation status switches occurred in borderline (*n* = 1; subtotal resection, completed Stupp protocol; progression-free survival (PFS) 23.7 months) and methylated *MGMT*p cases (*n* = 2; both gross total resections, completed Stupp protocol; PFS 28.3 and 52.3 months) to an unmethylated status on recurrence. It should be noted that one of the methylated *MGMT*p cases that switched to an unmethylated status on recurrence had a subsequent re-resection where re-testing demonstrated a switch back to a methylated status. No *MGMT*p switches occurred in those cases which were unmethylated on primary resection. 

The median *MGMT*p methylation percentage reduced in primary resections from 29.3% (range 3.3 to 76.8%; mean 28.1%) to 20.3% in recurrent resections (range 2.5 to 83.0%; mean 23.9%); however, this difference was not statistically significant (*p* = 0.41). 

Of the three cases with matched recurrence tissues only, one demonstrated an *MGMT*p switch from methylated to unmethylated in the VT block; however, interestingly, it maintained its *MGMT*p status in the NT block. 

## 4. Discussion

Here, we have demonstrated that necrosis levels in the majority of primary and recurrent HGGs do not influence *MGMT*p methylation status by pyrosequencing. When selecting tissue for molecular testing in solid tumors, pathologists are often directed to avoid areas of necrosis due to the assumption of a deleterious effect on DNA quality [38,44,45,46]. However, selecting a block without necrosis or with minimal necrosis is a particular challenge in high-grade gliomas, where necrosis is encountered in up to 95% of cases [39] and, in some subtypes, is a definitional feature [40]. 

There have been few prior studies that have quantified the effect of necrosis on the fidelity of molecular testing results in solid tumors. In non-small cell lung cancer, one study of 705 samples demonstrated that the presence or absence of necrosis did not influence the performance of an amplicon-based next-generation sequencing (NGS) assay [47], although the level of necrosis was not quantified. Similarly, in colorectal cancer, two studies evaluating the effect of necrosis on *KRAS* mutation detection found that a high content of necrosis, including in those specimens with up to 70% necrosis, had no effect on the results of either single-gene PCR or amplicon-based NGS assays [48,49]. These findings have been recently replicated in the validation of a targeted DNA/RNA-based NGS panel for a broader range of solid tumors (exclusive of primary CNS tumors), where necrosis was tolerated up to 100% [50]. These studies are limited in number but are in line with our current findings that necrosis does not have a negative effect on molecular testing as is often presumed. 

With respect to primary CNS tumors, the effect of necrosis on the molecular results is poorly characterized. In a study of solid tumor NGS panel testing, which included 43 primary CNS tumors, three of seven samples that failed library preparation were highly necrotic HGGs [51]. *MGMT*p methylation testing of 106 cases of primary GBM by MSP was performed, in which no association between *MGMT*p status and necrosis was found; however, the influence of necrosis on the reproducibility of the result was not assessed [52]. Our study is the first to quantify the effect of necrosis on *MGMT*p methylation testing, where we have established a threshold of minimum tumor cellularity (60%) at which necrosis does not appear to materially affect the replicability of *MGMT*p methylation testing.

We found that 84.6% of primary HGGs maintained their *MGMT*p status irrespective of the presence of necrosis. The majority of those with changed *MGMT*p status switched between borderline and methylated states (5 of 8), which in our local practice would not affect the clinical interpretation of the result. These cases had methylation percentages within 10% of our clinically validated borderline cut-off of 13%. Those that changed to an unmethylated status had more than 30% necrosis in the NT block and, again, were not highly methylated in the VT block (methylation percentages between 11 and 34.3%). It, therefore, appears that those cases with methylation percentages close to the borderline zone on VT block testing may be more susceptible to the influence of necrosis. 

While those primary HGGs that changed from methylated to unmethylated had >30% necrosis, it should be noted that those cases with maintenance of an unmethylated status harbored levels of necrosis between 5 and 90% in the NT blocks. This led us to postulate whether factors other than necrosis may affect *MGMT*p results, including the intratumoral heterogeneity of *MGMT*p methylation. A 2007 study of 25 cases of both GBMs and ‘anaplastic astrocytomas’, where multiple intratumoral biopsies were taken from each tumor, found intratumoral homogeneity in the *MGMT*p methylation status [53]. This is in contrast to a 2016 study of 14 GBM cases, where intraoperative sampling was conducted in a similar fashion, and intratumoral heterogeneity of *MGMT*p methylation and *MGMT* transcription was found [54]. The inclusion of three cases of ’molecular GBM’ yielded a finding that would be more in keeping with the latter study: while two of the three included ‘molecular GBM’ cases maintained their methylated status, one case changed from methylated to unmethylated on testing of a different block. While we cannot be certain of the role of intratumor heterogeneity in *MGMT*p methylation status, our findings indicate necrosis may not be the only element influencing *MGMT*p methylation status within a single tumor. This represents an interesting avenue for future investigation, with the potential for spatial transcriptomic analysis to resolve changing *MGMT*p methylation levels in the context of intratumoral heterogeneity.

In recurrent HGG, again, the majority of cases (78.3%) maintained their *MGMT*p status. Of those that changed *MGMT*p status with necrosis, two cases strangely showed increased methylation on re-testing of the NT block, with changes from unmethylated to methylated noted, including on repeat testing. Both cases had widely divergent degrees of necrosis in the NT block, each 5% and 60%. Interestingly, patients with *MGMT*p status changes all received and completed the Stupp protocol. Overall, it is difficult to make recommendations based on these small case numbers, except that there should be a low threshold for repeat testing using an alternative tissue block where an unexpected *MGMT*p methylation result is obtained in the setting of recurrence. 

Our evaluation of matched primary and recurrent HGGs is consistent with the previous literature; that is, the majority of HGG cases (81.3%) do not switch *MGMT*p status at recurrence. A 2019 meta-analysis of 18 studies, including 476 GBM patients with recurrence, found that *MGMT*p status switches in 24% of patients [55]. A more recent study of 40 GBM cases similarly showed unchanged *MGMT*p in 77.5% of cases [56]. With regards to changes in the *MGMT*p methylation percentage, there was a potential reduction from primary tumor to recurrence in our study, although this change did not reach statistical significance. Similarly, a study using the same pyrosequencing assay as we used, also documented a similar reduction, albeit not significant, from 20.35% to 14.25% (*p* = 0.346) [57]. It is noteworthy that all three patients who demonstrated an *MGMT*p status switch completed the Stupp protocol. Possible causes for an *MGMT*p status switch may include the evolution of TMZ-resistant subclonal populations; however, their influence on *MGMT*p status is still under investigation [58,59]. 

The limitations of our study include the overall sample size, as well as sample numbers in the sub-cohorts, i.e., GBM recurrence (*n* = 27) and cases that showed *MGMT*p switches (*n* = 16). An overall larger cohort of GBM patients would, by extension, increase numbers within these sub-cohorts. This would allow for more definitive conclusions regarding the effect of necrosis on *MGMT*p status at recurrence, as well as *MGMT*p switches at recurrences. Another limitation of our study is the deliberate selection of methylated cases over unmethylated cases. This was necessary for our study design so that the effect on necrosis could be evaluated on the result of *MGMT*p methylation in the laboratory setting. However, from a clinical perspective, this skewed the GBM patient cohort [24] and precluded survival analyses. Again, a larger representative cohort with a greater frequency of *MGMT*p unmethylated patients is needed. Lastly, *MGMT*p cut-off values by pyrosequencing may vary between laboratories due to differences in assay methodology and validation protocols. However, our study demonstrates that the presence of tumor necrosis does not significantly impact the percentage of *MGMT*p methylation. Again, a larger multi-institutional cohort would help resolve and standardize *MGMT*p methylation percentages between institutions.

## 5. Conclusions

The words attributed to Mark Twain—‘*The reports of my death are greatly exaggerated*’—ring true when examining the effects of necrosis on *MGMT*p methylation testing in HGGs. We demonstrate that the majority of primary and recurrent HGGs maintain their *MGMT*p status in the presence of necrosis and have established a tumor cellularity cut-off (60%) at which necrosis does not affect *MGMT*p status calling. We also identify scenarios in which caution must be exercised in the interpretation of *MGMT*p testing, including borderline results and recurrent HGG, where a low threshold for repeat testing is recommended. Switches in *MGMT*p status between primary and recurrent HGGs were not observed in most cases, in keeping with previous studies. Further investigation of the role of intratumoral heterogeneity, particularly in the setting of ‘molecular GBMs’ and treatment-induced necrosis, will deepen our understanding of the effects of *MGMT*p methylation on clinical outcomes. 

## Figures and Tables

**Table 1:** Summary of patient characteristics.

	All Patients (*n* = 64)	
**Age at diagnosis (years)**		
Median	60	
Range	31 to 86	
**Gender**	*n*	%
Male	40	62.5
Female	24	37.5
**Integrated WHO diagnosis ^1^**		
GBM, IDHwt	57	89.1
Astro IDHm G4	2	3.1
DHG H3 G34	1	1.6
‘molecular GBM’	4	6.3
**Primary tumor**	**58**	90.6
Gross total resection	46	71.9
Partial/subtotal resection/biopsy	12	18.6
Chemotherapy	56	87.5
Radiotherapy (60 Gy or 40 Gy)	47	73.4
Stupp protocol completed	35	54.7
**Recurrent tumor**	**23**	37.5
Matched primary	16	25.0
Unmatched	7	12.5
Gross total resection	17	32.8
Partial/subtotal resection/biopsy	6	9.8

^1^ GBM IDHwt = glioblastoma, IDH-wildtype, CNS WHO grade 4; Astro IDHm G4 = astrocytoma, IDH-mutant, CNS WHO grade 4; DHG H3 G34 = diffuse hemispheric glioma, H3 G34-mutant, CNS WHO grade 4; ‘molecular GBM’ = diffuse astrocytic gliomas, histologically grade 3 with molecular feature of GBM (*TERT* promoter variant).

**Table 2:** Summary of primary and recurrent presentations of HGGs with maintenance of *MGMT*p status.

*MGMT*p Status	Viable Tumor Block (VT)	Necrotic Tumor Block (NT)	*p*-Value
***Primary presentation***	*Median %, (range)*	*Median %, (range)*	
Methylated (*n* = 35)	36.8 (14–92.5)	42.3 (14.5–83.8)	0.93
Borderline (*n* = 3)	11.3 (9.8–12.5)	10.5 (9.3–12.5)	0.82
Unmethylated (*n* = 6)	4 (3.3–5.5)	3.4 (2.3–4)	0.07
***Recurrent presentation***	*Median %, (range)*	*Median %, (range)*	
Methylated (*n* = 8)	30.3 (19.5–83)	34.7 (24.5–73.5)	0.57
Unmethylated (*n* = 10)	3.4 (2.5–5.8)	3.6 (2.3–6.5)	0.97

**Table 3:** Changes in *MGMT*p status by degree of necrosis in NT block.

Degree of Necrosis (%)	Primary Cases (*n*)	Primary *MGMT*p Status Changes (*n*)	Changes ^1^	Recurrence Cases (*n*)	Recurrence *MGMT*p Changes (*n*)	Changes ^1^
90	2	0	-	0	0	-
80	1	1	M→U *	1	1	M→U *
70	2	1	M→B	3	0	-
60	5	0	-	2	1	U→M *
50	8	2	M→B B→U *	2	0	-
40	6	0	-	0	0	-
30	5	1	B→U *	3	0	-
20	8	0	-	2	1	M→U *
≤10	15	3	B→M M→B M→B	10	2	M→B U→M *

^1^ M = methylated; B = borderline; U = unmethylated; → indicates change; ‘*’ denotes *MGMT*p status switches of potential clinical significance.

## Data Availability

The original data presented in the study are openly available in the Supplementary Materials.

## Supplementary Materials

The following supporting information can be downloaded at: https://www.mdpi.com/article/10.3390/cancers16101906/s1, Table S1: Cohort characteristics.
